# Krüppel-Like Factor 6 Expression Changes during Trophoblast Syncytialization and Transactivates *ßhCG* and *PSG* Placental Genes

**DOI:** 10.1371/journal.pone.0022438

**Published:** 2011-07-22

**Authors:** Ana C. Racca, Soledad A. Camolotto, Magali E. Ridano, José L. Bocco, Susana Genti-Raimondi, Graciela M. Panzetta-Dutari

**Affiliations:** Centro de Investigaciones en Bioquímica Clínica e Inmunología (CIBICI-CONICET), Departamento de Bioquímica Clínica, Facultad de Ciencias Químicas, Universidad Nacional de Córdoba, Córdoba, Argentina; Institute of Zoology, Chinese Academy of Sciences, China

## Abstract

**Background:**

Krüppel-like factor-6 (KLF6) is a widely expressed member of the Sp1/KLF family of transcriptional regulators involved in differentiation, cell cycle control and proliferation in several cell systems. Even though the highest expression level of KLF6 has been detected in human and mice placenta, its function in trophoblast physiology is still unknown.

**Methodology/Principal Findings:**

Herein, we explored KLF6 expression and sub-cellular distribution in human trophoblast cells differentiating into the syncytial pathway, and its role in the regulation of genes associated with placental development and pregnancy maintenance. Confocal immunofluorescence microscopy demonstrated that KLF6 is expressed throughout human cytotrophoblast differentiation showing no evident modifications in its nuclear and cytoplasmic localization pattern. KLF6 transcript and protein peaked early during the syncytialization process as determined by qRT-PCR and western blot assays. Overexpression of KLF6 in trophoblast-derived JEG-3 cells showed a preferential nuclear signal correlating with enhanced expression of human β-chorionic gonadotropin (*βhCG*) and pregnancy-specific glycoprotein (*PSG*) genes. Moreover, KLF6 transactivated *βhCG5*, *PSG5* and *PSG3* gene promoters. Deletion of KLF6 Zn-finger DNA binding domain or mutation of the consensus KLF6 binding site abolished transactivation of the *PSG5* promoter.

**Conclusions/Significance:**

Results are consistent with KLF6 playing a role as transcriptional regulator of relevant genes for placental differentiation and physiology such as *βhCG* and *PSG*, in agreement with an early and transient increase of KLF6 expression during trophoblast syncytialization.

## Introduction

Trophoblast is the first cell lineage to differentiate during mammalian development. They are the precursor cells of the human placenta, mediate implantation and give rise to most of the extraembryonic tissues. After the initial phase of nidation, human cytotrophoblast (CTB) cells differentiate along two pathways: the invasive pathway to become extravillous trophoblasts or the syncytial pathway to become villous trophoblasts. Extravillous trophoblasts invade deep into the uterus wall and are directly implicated in anchoring the chorionic villi in the uterus. The villous CTB cells in the syncytial pathway fuse and differentiate into the syncytiotrophoblast (STB) layer, which is a multinucleated epithelium-like surface maintained by continuous cell fusion of the underlying mononuclear CTBs. The STB plays a major role throughout pregnancy, as it is the primary site for maternal–fetal exchange of nutrients, gas, and waste products, and for the synthesis of hormones required for fetal growth and development (reviewed in [1]). Placenta development and trophoblast differentiation are associated with the up-regulation of a number of genes regarded as pregnancy specific; among them are the *hCG* and *PSG* gene families and the glial cell missing homolog 1 (*GCM1*) gene which encodes a transcription factor essential for STB differentiation [2]. hCG has a central role in the formation of human placental syncytium and pregnancy maintenance, indeed aberrant serum levels are largely used to monitor pregnancy-associated complications [3], [4]. Multiple variants of hCG have been described with relevant functions in the villous CTB and STB cells, as well as in the extravillous invasive CTB cells [5]. hCG is composed of a specific β-subunit and an α-subunit common to FSH, LH, and TSH [5]. Synthesis of the β-subunit is associated with fusion of CTBs into the multinuclear syncytium. It is encoded by the *βhCG* gene cluster, which comprises six members arranged on chromosome 19, being *βhCG5* gene the most abundantly transcribed in choriocarcinoma cells and placenta [4]. The human *PSG* family is composed of 11 similar genes, also arranged on chromosome 19, whose expression is markedly induced upon early trophoblast differentiation. They encode for the most abundant placental proteins found in the maternal circulation in late pregnancy and are also essential for maintenance of gestation [6], [7]. Although *PSG* genes share more than 93% nucleotide sequence identity, some of them are highly expressed (i.e. *PSG1*, *PSG3* and *PSG5*) while others remain low-expressed in STB cells [7].

KLF6 is a member of the Krüppel-like transcription factor family which has been independently cloned from placenta [8], leukocyte [9] and liver [10] cDNA libraries. KLF6 is evolutionarily conserved and broadly expressed in numerous cell types and at several developmental stages, although its transcript is enriched in liver, prostate, lung and placenta [8], [11], [12], [13], [14], [15]. This protein has three C_2_H_2_ zinc fingers at its C-terminal domain, responsible for specific DNA-binding to either GT/GC-box or CACC-element sites on responsive promoters. Experiments in several cell systems have revealed a general and sometimes dissimilar role for KLF6 in the regulation of cellular events as proliferation, differentiation and apoptosis [16]. KLF6 has been mainly implicated as a tumor suppressor gene, frequently inactivated in a variety of human cancers (reviewed in [17]). Nevertheless, several other large studies established that genetic alterations of KLF6 are rarely found in distinct types of human cancers. Moreover, it has been reported that KLF6 gene expression was enhanced or its sub-cellular localization was modified in some tumors [18], [19], [20], [21]. Current available information strongly suggests that the final outcome of KLF6 function depends on the particular cell environment, biochemical signaling, interaction with specific transcriptional partners and/or its sub-cellular distribution [21].

*Klf6−/−* knockout mice die by embryonic day 12.5 and are characterized by reduced hematopoietic differentiation in yolk sacs and impaired placental development [22]. Despite the fact that KLF6 is highly expressed in human and mice placenta tissue [8], [12], [23], little is known about its expression during villous trophoblast cell differentiation and about its role in the transcription of placenta specific genes.

The aim of this work was to explore KLF6 expression in human trophoblast cells undergoing differentiation into the syncytial pathway, and to provide evidence for its role as a transcriptional regulator of genes involved in placenta and maintenance of pregnancy.

## Results

### KLF6 protein is expressed throughout the CTB differentiation into the syncytial pathway

**Figure 1:**
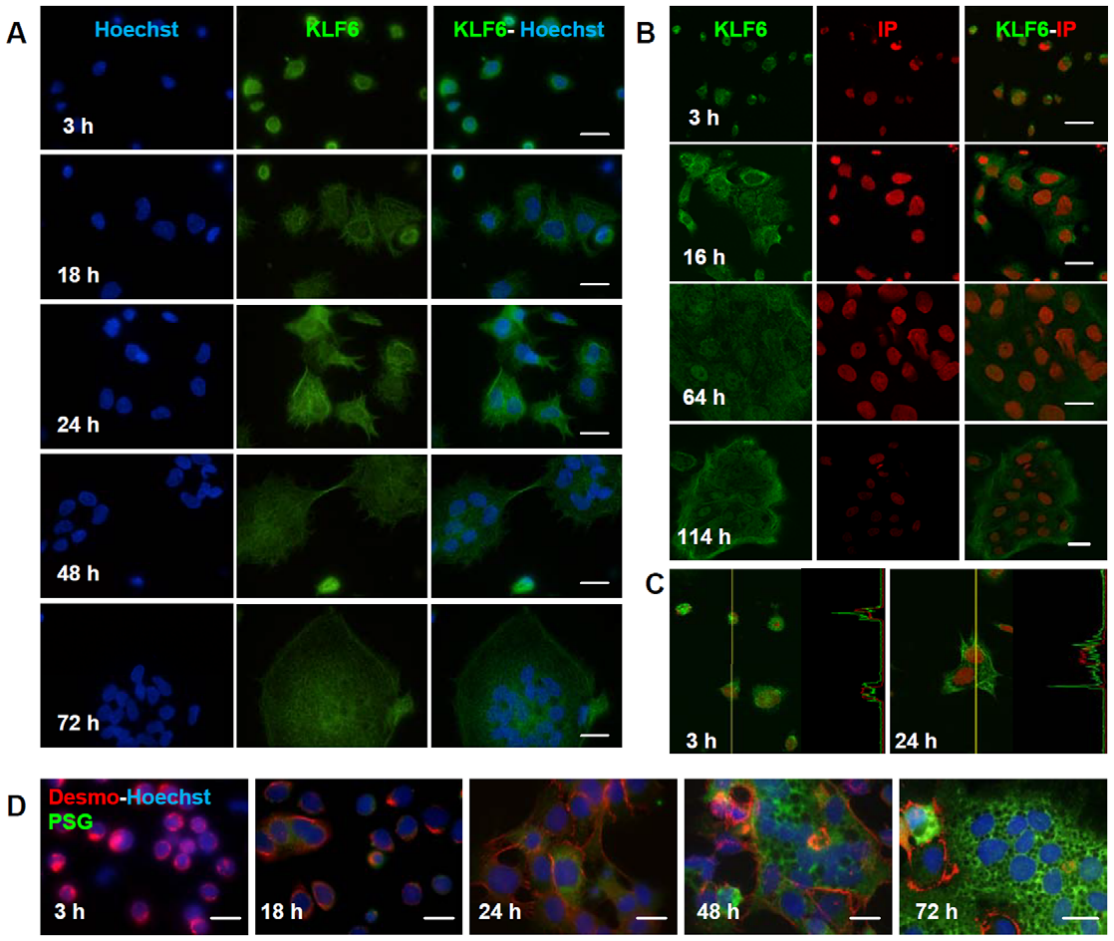
KLF6 protein expression throughout trophoblast cell differentiation. **A-** Isolated mononuclear villous CTB cells cultured during the indicated hours and stained for KLF6 immunofluorescence detection (middle panels) with the polyclonal R-173 (green) anti-KLF6 antibody. Nuclei were counterstained with Hoechst 33342 dye (blue) and the overlay is shown (right panels). **B-** Confocal microscopy imaging of KLF6 at the indicated time points of the differentiation process. KLF6 was labelled with the polyclonal R-173 antibody (left panels) and DNA was stained with propidium iodide (IP) (middle panel). Overlay is shown in the right panels. **C-** Fluorescence intensity profile of KLF6 (green) and IP (red) along the yellow line shown in the confocal microscopy images. **D-** Morphological and biochemical differentiation of isolated mononuclear CTB cells were confirmed by the disappearance of desmoplakin intercellular staining (red), the appearance of multinucleated structures and the expression of PSG proteins (green). Original magnification, x1000. Scale bar, 10 µm. Immunofluorescence assays were performed with at least three different CTB purifications and representative figures are shown.

KLF6 has been detected in villous trophoblast cells with a nuclear and cytoplasmic signal, [21], [23] however, it is not known whether KLF6 is expressed through the whole syncytialization process and if it maintains the same nucleo-cytoplasmic pattern. Therefore, CTB cells were isolated from human normal-term placenta and cultured, as previously described [7]. KLF6 protein was detected during the entire differentiation process by immunofluorescence assays, as visualized by conventional epifluorescence microscopy (Fig 1A). Confocal immunofluorescence microscopy revealed a cytoplasmic as well as nuclear staining in CTB cells at several time points of the differentiation process (Fig 1B). The presence of KLF6 in the nucleus was clearly observed performing fluorescence intensity profiles where the green peak (KLF6 signal) matches with the red peak (nuclear signal) (Fig 1C). Morphological and biochemical differentiation of CTBs into the STB pathway was corroborated by fluorescence staining for nuclei, desmosomes and PSG proteins (Fig 1D), as well as by the increase in PSG and βhCG mRNA levels (data not shown). In sum, these results established that KLF6 is expressed throughout the differentiation of CTB cells into the syncytial pathway and that it is localized to both the nucleus and the cytoplasm during this process.

### Early and transient increase of KLF6 transcript and protein levels during CTB cell differentiation

**Figure 2:**
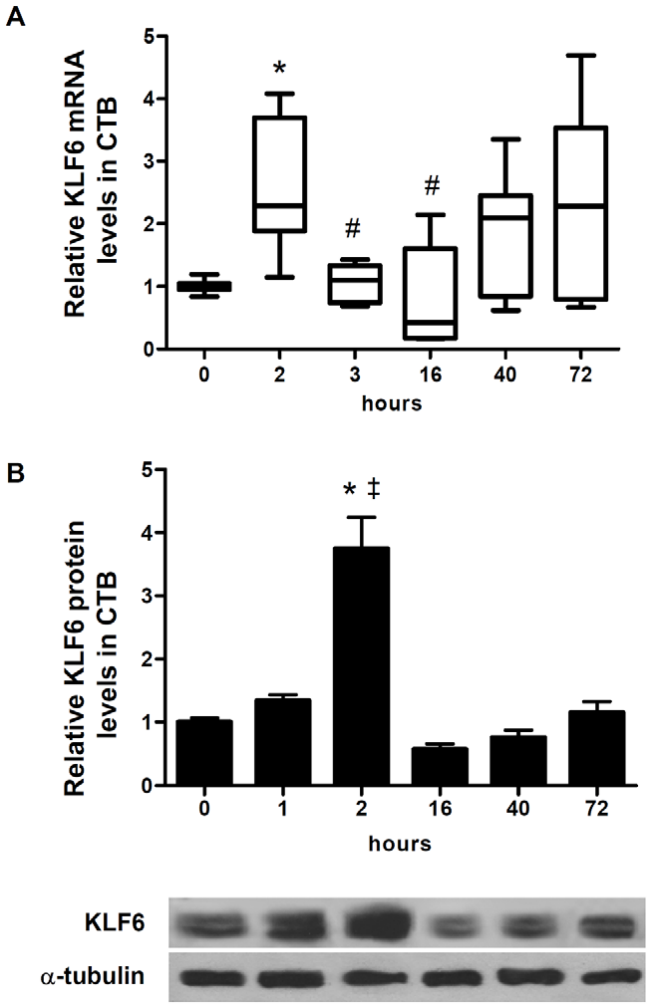
KLF6 transcript and protein levels increase during trophoblast cell differentiation. **A-** KLF6 mRNA expression was quantified by qRT-PCR (ABI 7500, Applied Biosystems) in CTB cells isolated from three to eight normal term placentas, and cultured in differentiating medium during the indicated times. Results were normalized to cyclophilin A and expressed according to the 2^−ΔΔCt^ method using as calibrator the expression level at 0 h. Results are depicted as boxplot graphs representing the medians (horizontal bars), the 25–75th percentile interquartile range (box limits), and the lowest and highest values (whiskers) of three to eight experiments performed in triplicates. Inter-group comparisons were made using the Kruskal-Wallis one-way Analysis of variance (ANOVA) with the Dunn's multiple comparisons post-hoc test of statistical significance. *p<0.05 *vs* 0 h, #p<0.05 *vs* 2 h. **B-** Protein extracts (60 µg) prepared from CTB cells cultured for the indicated hours were subjected to western blot analysis using anti-KLF6 and α-tubulin antibodies as described in *Materials and Methods*. A representative blot is shown. The bar graph represents the densitometric quantification of KLF6/α-tubulin ratio of three independent experiments expressed as mean ±SEM. *p<0.05 *vs* 0 h, ‡p<0.05 *vs* 16 h. (Kruskal-Wallis, Dunn's).

KLF6 transcript expression was evaluated by qRT-PCR assays at different times during the *in vitro* CTB differentiation process. KLF6 mRNA was detected in freshly isolated CTBs, its expression was induced more than 2-fold after 2 h of differentiation, then decreased over the next 16 h, increasing then again by an amount dependent on the placenta evaluated (Fig 2A). Furthermore, KLF6 protein was also early induced during CTB differentiation reaching a peak at 2 h that was near 3.5 fold greater than the baseline levels and decreased by 80% over the next 16 h, increasing again from that point with a similar kinetic to that observed for mRNA (Fig 2B). Altogether these data indicate that KLF6 expression is regulated during syncytialization, exhibiting an early and transient increase followed by a less consistent increase towards 72 h of differentiation, where syncytium-like structures were clearly formed. These results suggest that KLF6 may be involved in the transcriptional control of genes associated with or expressed in syncytial trophoblast.

### KLF6 induces mRNA and protein expression of trophoblast differentiation marker genes

**Figure 3:**
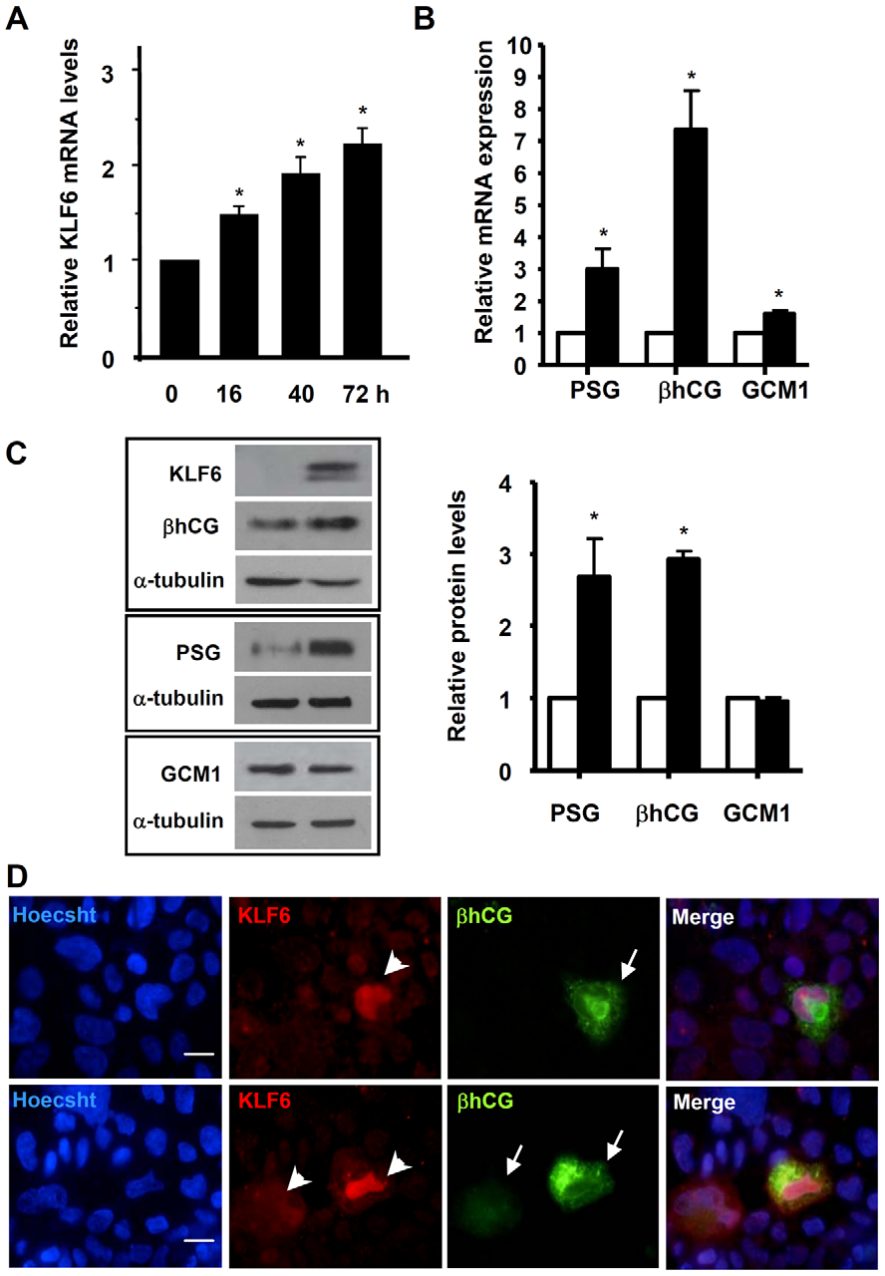
KLF6 regulates mRNA and protein expression of placental genes. **A-** JEG-3 cells were cultured in the presence of 1 µM methotrexate to induce cell differentiation during the indicted times and KLF6 mRNA expression was quantified by qRT-PCR (ABI 7500, Applied Biosystems). **B-** JEG-3 cells transfected with the empty (white bars) or the KLF6 expression vector (black bars) were harvested 24 h after transfection and *PSG*, *βhCG* and *GCM1* gene expression was quantified by qRT- PCR (ABI 7500, Applied Biosystems). For A and B, results were normalized to cyclophilin A and expressed according to the 2^−ΔΔCt^ method using as calibrator the mRNA level obtained from the control condition. Data are presented as mean ±SEM of three independent experiments performed in triplicates and a one-sample t-test was used to determine whether experimental values were significantly different from the control value set as 1 (*p<0.05). **C-** Western blot detection of KLF6, PSG, βhCG and GCM1 in protein extracts of JEG-3 cells transfected with the empty (left lane) or KLF6 expression vector (right lane). α-tubulin was used as a loading control in each assay. Representative western blots are shown and the bar graph shows the densitometric analysis of three different experiments. (*p<0.05) **D**- JEG-3 cells were transiently transfected with the KLF6 expression vector and 24 h later they were immunostained for the detection of KLF6 (red) and βhCG (green) with a monoclonal anti-KLF6 and a polyclonal anti-βhCG antibodies, respectively. Nuclei were counterstained with Hoechst 33342 dye (blue), and the merge of the three channels is shown on the right side. Bar = 10 µm. Original magnification: ×1000. Representative images from three independent transfections are shown. Arrowheads, JEG-3 cells overexpressing KLF6; arrows, cells positive for βhCG.

There is a growing list of KLF6 target genes involved in cellular growth, differentiation, adhesion and endothelial motility, as well as metabolism and inflammation [17]. Although KLF6 was isolated by target site screening of a placental expression library using a *PSG5* gene promoter element as a probe [8], there is limited evidence demonstrating that KLF6 indeed regulates *PSG* or other placental relevant genes in trophoblastic cells. The trophoblast-related JEG-3 cell line was selected to analyze the role of KLF6 as transcriptional regulator of trophoblastic genes. We considered this cell line a suitable model because in normal culture conditions they express low levels of *PSG* and *βhCG*. In addition, they form syncytium-like structures and up-regulate the expression of both genes when they are cultured in the presence of methotrexate [7]. Furthermore, herein we demonstrate that KLF6 basal expression is low in JEG-3 cells but it is also increased when they are stimulated to differentiate with methotrexate (Fig 3A). Thus, JEG-3 cells were transiently transfected with the pXJ-41-KLF6 expression vector and the mRNA and protein levels of relevant placental genes were evaluated 24 hours later. KLF6 overexpression induced an increase in PSG and βhCG mRNA levels near 3- and 8-fold over the control, respectively (Fig. 3B). In addition, the expression of the GCM1 transcription factor was slightly but significantly stimulated (1.5-fold, p<0.05). Western blot analysis showed that PSG and βhCG protein levels were significantly higher in transfected cells relative to control cells, while GCM1 protein expression remained unmodified (Fig. 3C).

KLF6 effect on the activation of βhCG synthesis was further demonstrated by co-immunofluorescense assays of JEG-3 cells transiently transfected with the pXJ-41-KLF6 expression plasmid. Protein overexpression and nuclear localization of KLF6 were confirmed by western blot and immunofluorescence assays, respectively (Fig. 3C & D). As depicted in figure 3D, transfected cells showed a clear increase in KLF6 signal (arrowheads) compared with untransfected cells where a very low signal was detected. Accordingly, cells clearly positive for βhCG (arrows) were those overexpressing KLF6. In sum, these results provide strong evidence for a role of KLF6 as a regulator of important syncytium-related genes.

### KLF6 transactivates *βhCG* and *PSG* promoters in trophoblastic cells

**Figure 4:**
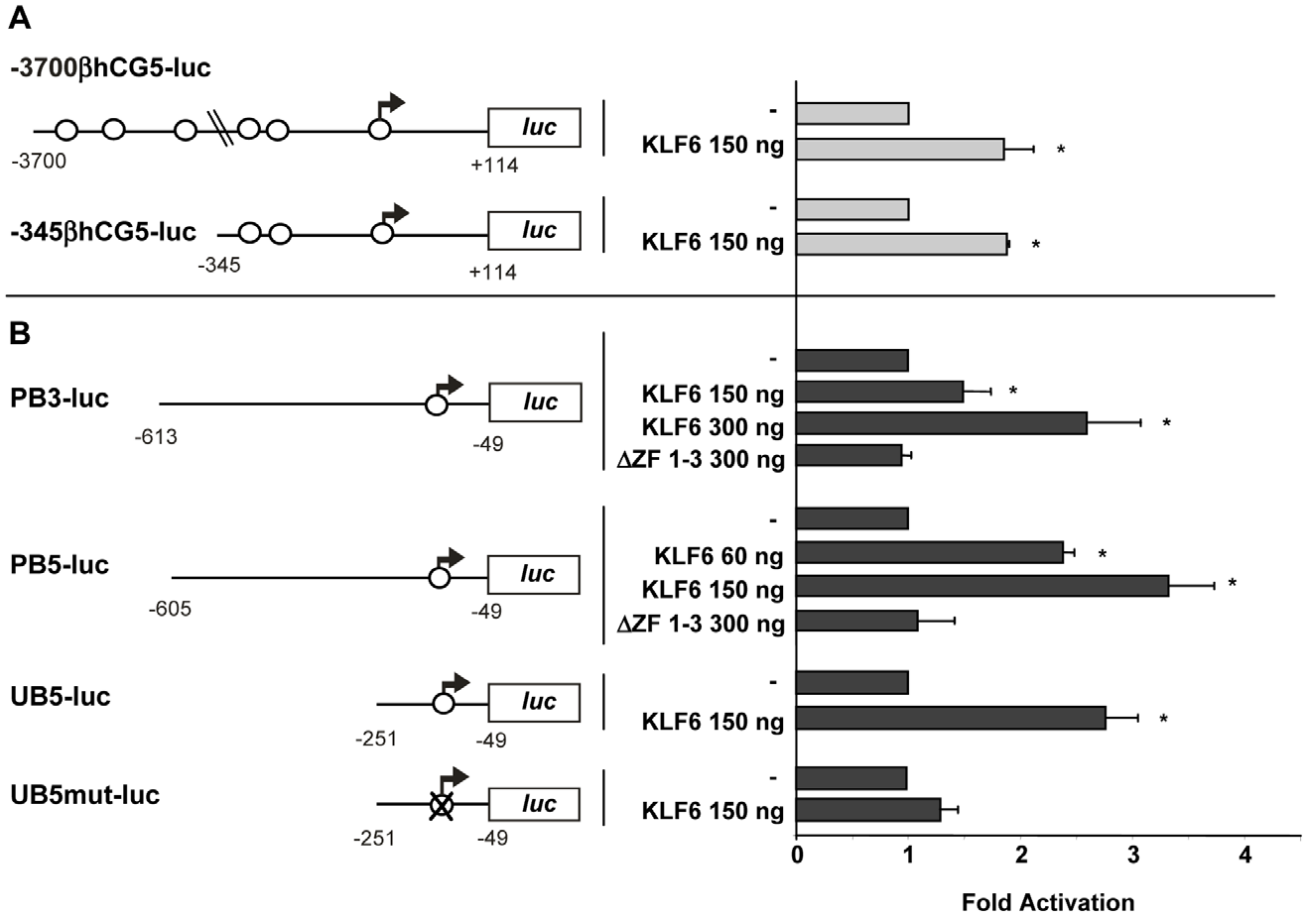
KLF6 transactivates *βhCG, PSG5* and *PSG3* genes in human trophoblastic cells. The human wild-type KLF6 (KLF6) expression plasmid, the Zn-finger deleted KLF6 (ΔZF 1–3) construct or the empty (-) expression vector were cotransfected into JEG-3 cells together with the *Renilla* reporter plasmid and (**A**) the human *βhCG5* promoter luciferase reporter plasmids: −3700βhCG5-luc and −345βhCG5-luc or (**B**) the *PSG5* or *PSG3* promoter constructs: PB5-luc, PB3-luc, UB5-luc and UB5mut-luc, as depicted on the left. *PSG5* and *PSG3* consensus KLF6 binding sites, as well as putative KLF6 binding motifs in the *βhCG5* gene are depicted as circles. Luciferase activity was normalized to *Renilla* activity. The KLF6 expression plasmid quantity used is indicated. Results are expressed as fold activation respect to each promoter construct co-transfected with the empty vector. Data from three or four experiments performed in duplicate are shown (mean ±SD). * Statistically significant difference (p<0.05) between the fold activation of each construct in the presence respect to the absence of the expression vectors is shown.

To analyze whether the induction of βhCG and PSG mRNA levels by KLF6 involves the transcriptional activation of their promoters, JEG-3 cells were co-transfected with the KLF6 expression plasmid and different *βhCG5*, *PSG3* and *PSG5* promoter constructs coupled to a luciferase reporter gene. Reporter activity of the promoter constructs containing nt −3700/+114 and −345/+114 of *βhCG* was increased in KLF6 overexpressing cells compared to cells transfected with the empty expression vector (Fig. 4A). No differences were found in fold activation between the short (1.9±0.1 fold, p<0.01) and long (1.8±0.2 fold, p<0.01) *βhCG5* promoter constructs. Therefore, KLF6 is able to transactivate the *βhCG5* gene through sequences present in the −345/+114 promoter region.

We have previously demonstrated that in non-placental COS-7 cells, *PSG5* minimal promoter construct activity depends on a core promoter element (CPE) recognized *in vitro* by a recombinant KLF6-βgalactosidase fusion protein, and this activity was slightly induced by overexpressing KLF6 [8], [24], [25]. Herein, we analyzed whether KLF6 is a transcriptional activator of the *PSG5* and *PSG3* promoters in trophoblastic cells. Both genes share near 94% identity in their promoter sequences and their KLF6 consensus binding motifs (CPE) differ in two nucleotides, one in the core and the other in the surrounding sequence. The PB5-luc (PSG5 nt −605/−49) and the PB3-luc (PSG3 nt −613/−49) promoter constructs were activated by KLF6 overexpression in a dose-dependent manner (Fig. 4B). In the presence of 150 ng of the expression vector PB5-luc was activated 3.3±0.4 fold, while the PB3-luc construct was activated 1.5±0.2 fold. Co-transfection experiments performed with a KLF6 expression vector lacking the three zinc-finger domains revealed that the KLF6 DNA binding domain is required for *PSG3* and *PSG5* promoter transactivation (Fig. 4B lanes ΔZF1-3). In addition, KLF6 also enhanced the promoter activity of a shorter PSG5 regulatory region (nt −251/−49) and this transactivation was markedly reduced when the CPE consensus motif was mutated (Fig. 4B, UB5-luc compared to UB5mut-luc).

These results support the role of KLF6 as a regulator of *PSG* and *βhCG* gene transcription in the context of trophoblast cells. Additionally, they indicate that *PSG* gene transactivation requires the KLF6 DNA binding domain as well as the wild type CPE sequence, at least for the *PSG5* gene.

## Discussion

This study demonstrates that KLF6 is present in the nucleus and cytoplasm of trophoblast cells throughout the syncytialization process; KLF6 expression shows an early and transient increase during differentiation, and it functions as a transcriptional activator of *βhCG5*, *PSG5* and *PSG3* genes in a trophoblastic cell line. In addition, the KLF6 Zn-finger DNA binding domain is required for *PSG* gene transactivation.

Although KLF6 primary amino acid sequence analysis reveals a conserved nuclear localization signal and predicts its nuclear localization, KLF6 is frequently detected in the cytoplasm of several tissues and cell lines [21]. KLF6-SV1 is a splice variant which encodes for a truncated KLF6 protein with a novel C-terminal domain mainly associated with a cytoplasmic localization [26], [27]. Thus, it is possible to postulate that the KLF6 cytoplasmic signal observed in trophoblast cells could be the protein product of this spliced variant. However, KLF6-SV1 mRNA and protein were not detected in trophoblastic cells by semiquantitative PCR or western blot assays, respectively (data not shown), suggesting that it is not the main source of cytoplasmic KLF6 in placenta. It has been reported that KLF6 is relocated from the cytoplasm to the nucleus in response to different stimuli. For instance, KLF6 was found to be distributed in the cytoplasm of human umbilical vein endothelial cells and was shifted from the cytoplasm to nuclei after injury followed by the transactivation of the endoglin promoter [28]. Even though we cannot rule out an enrichment of KLF6 within the cell nucleus at some point of the syncytialization pathway, KLF6 was indeed located in the nucleus of trophoblast cells throughout the differentiation process allowing it to function as a transcription factor. In addition, it is possible to postulate that KLF6 may participate in other biological processes in the cytoplasm. This hypothesis is in line with recent findings which demonstrate that cytoplasmic KLF6 is able to interact with c-Src protein and thereby to interfere with estrogen receptor alpha-mediated cell growth of breast cancer cells [29].

It has been previously demonstrated that *KLF6* is co-expressed with *PSG* in membranes and placenta tissues suggesting a function as a regulator of *PSG* gene transcription *in-vivo* [23]. Herein, we provide evidence for a role of KLF6 as transcriptional activator not only of *PSG3* and *5* genes but also of *βhCG5* gene promoters in trophoblastic cells. Although we have not investigated the molecular mechanism involved, present results indicate that, at least for *PSG5*, transactivation was dependent on the KLF6 Zn-finger C-terminal domain and the CPE-box, a GC-rich element, present in the *PSG* promoters. Results are compatible with KLF6 acting as a nuclear DNA-binding transcription factor. However, the ΔZF1-3 KLF6 construct employed in our studies lacks the Zn-finger domain and its 5′ adjacent sequence, which was recently demonstrated to be required for KLF6 translocation to the nucleus [27]. Therefore, we cannot discard the possibility that KLF6 may transactivate the *PSG* and *βhCG* promoters not through the direct binding to its consensus sites, but through the association with other transcription factors recruiting KLF6 to the regulatory region of their target genes. In support of this hypothesis, *PSG5* and *βhCG5* promoters are also regulated by the Sp1 transcription factor [4], [30] and additionally, functional cooperation between KLF6 and Sp1 is important for the transcriptional regulation of some common target genes. This cooperation involves the physical interaction between Sp1 C-terminal transactivating domain and KLF6 N-terminal DNA binding domain [28], [31]. Thus, in the nucleus, KLF6 might directly recognize its binding site and/or interact with Sp1 enhancing *PSG* and *βhCG* gene transcription.

Transient overexpression of KLF6 stimulated endogenous PSG and βhCG mRNAs and induced a marked increase in their protein levels, emphasizing KLF6 contribution to the activation of *PSG* and *βhCG* expression in trophoblastic cells. In addition, KLF6 overexpression lead to a slight but significant increase in the endogenous GCM1 mRNA with no apparent effect at the protein level. The absence of an absolute correlation between the induction of PSG, βhCG and GCM1 mRNA levels and their corresponding protein levels might reflect differences in their mRNA and protein half lives. This finding is also in line with the contribution of post-transcriptional mechanisms involved in the control of these trophoblast markers. Indeed, *βhCG* and *GCM1* expression is regulated at both the transcriptional and translational level including ubiquitination and proteasome-mediated degradation in mice and human trophoblast cells [32], [33], [34].

KLF6 mRNA and protein levels change throughout syncytialization indicating that its gene expression is regulated during this process. To our knowledge this is the first report indicating an increase in KLF6 expression associated to trophoblast differentiation into the syncytial pathway and opens up an avenue for future research on the mechanisms implicated. In other cell models, the expression of KLF6 is enhanced by Sp1, IGF-I and TGF-β1 [15], [31], [35]; and KLF6 protein stability is controlled by the JNK and p38 pathways, as well as by its nucleo-cytoplasmic localization domains [27], [36].

KLF6 has been largely associated with tumor biology [16]. Nevertheless, accumulating evidence indicates that KLF6 may be a general regulator of cell differentiation and organ development. In this sense, KLF6 expression is developmentally regulated in the mouse cornea, lens and during preadipocyte differentiation [37], [38], [39]. Furthermore, studies in *klf6*-/- mice and embryonic stem cells (ES) established that Klf6 promotes ES-cell proliferation and differentiation in early stages. Subsequently, it contributes to differentiation into mesoderm cells and finally, it plays a role in both hematopoiesis and vasculogenesis [22]. In addition, it has been recently demonstrated that Klf6 is essential for the development of endoderm-derived organs and for hepatocyte specification in mouse ES cells [40]. According to these published data and to the findings described here, it is attractive to propose that KLF6 participates not only in the differentiation of cells derived from the epiblast lineage but also from the trophoectoderm. We are currently addressing the role of KLF6 in trophoblast differentiation.

In conclusion, present results support a functional role for KLF6 in the regulation of placenta-specific marker genes, provide the first direct evidence for a regulated expression of KLF6 during CTB cell differentiation into the syncytial pathway, and reveal a nuclear as well as cytoplasmic localization during this differentiation process. Altogether, they support a role of KLF6 in human placental development and function, and are in good agreement with the impaired placental development found in *Klf6-/-* knockout mice [22]. Finally, these findings provide the basis for further studies to elucidate KLF6 function within the trophoblast environment in order to understand the precise processes this transcription factor modulates in the human placenta, where its expression is highly enriched.

## Materials and Methods

### Ethics statement

This study was conducted with the ethics approval from the Human Studies Committee of the Hospital Privado of Córdoba, Argentina (HP 4–112). Normal term placentas were collected from unidentified anonymous patients. Therefore, and because term placentas obtained after delivery were considered waste material and were normally discarded, informed consent was not required by the local ethic committee. Our IRB specifically waived the need for consent.

### Cytotrophoblast isolation, cell culture and differentiation

Tissues from normal term placentas (37–41 wk of pregnancy) were obtained after cesarean delivery and processed within 30 min after delivery. After removal of the cord, amniochorion, and decidual layer, villous tissue free of visible infarct, calcification or haematoma was sampled from the maternal-fetal interface. The tissue was cut into small pieces and washed with 154 mmol/L NaCl, to remove blood. CTBs were isolated according to the protocol of Kliman [41], with the modifications previously described [42]. Isolated trophoblasts (97% cytokeratin 7-positive cells) were plated in keratinocyte growth medium (KGM, Invitrogen) supplemented with 10% fetal bovine serum (FBS) and antibiotics (100 U/mL penicillin/0.1 mg/mL streptomycin). Culture medium was supplemented with 5 ng/mL of recombinant human Epidermal Growth Factor (EGF, Invitrogen, Cat. No. 10450-013) to enhance syncytialization [43], and cells were maintained in culture for up to 114 h with complete medium changed every 24 h. JEG-3 cells were grown in Dulbecco's modified Eagle's medium supplemented with antibiotics and 10% FBS. When required, they were cultured in the presence of 1 mM methotrexate to induce *in vitro* differentiation as described [7].

### Immunofluorescence assays

Isolated CTB and JEG-3 cells were cultured on cover slips using supplemented medium as described above. The cover slips were washed three times with PBS and cells were fixed 20 min in 3% paraformaldehyde at room temperature or 10 min in methanol at −20°C according to the antibody used. Immediately after, cells were incubated 10 min with 10 mM ammonium chloride to inhibit quenching, washed with PBS three times and permeabilized for 20 min with 0.5% Nonidet P-40 in PBS or 7 min with 0.1% Triton 100X in PBS. Cells were then rinsed with PBS three times, blocked with 2.5% normal goat serum in 0.2% Tween-20 in PBS (PBST) and with 0.5% fish skin gelatin in PBST, and then incubated with the following primary antibodies: rabbit polyclonal anti-KLF6 (anti-Zf9, R-173, Santa Cruz Biotech; 1∶50), mouse monoclonal anti-KLF6 (clone 2c11, 1∶150) whose specificity was previously determined [36], mouse anti-desmosomal protein (0.045 mg/mL, ZK-31, Sigma Chemical Co.; 1∶400), rabbit polyclonal anti-human chorionic gonadotropin (hCG, A0231, Dako; 1∶500), mouse monoclonal anti-cytokeratin 7 (Dako, Clone OV-TL 12/30) and rabbit polyclonal anti-PSG (A0131, Dako; 1∶100). Cells were washed with PBST, blocked as described above, and incubated with the appropriate species-specific secondary antibodies, either red Alexa Fluor 594-conjugated goat anti-mouse IgG (Fab) or green Alexa Fluor 488-conjugated donkey anti-rabbit IgG (Fab) (Molecular Probes) in a 1∶720 final dilution. Negative controls were performed replacing the primary antibodies with buffer. These controls ruled out nonspecific staining of CTB and JEG-3 cells by the secondary antibodies used (not shown). All antibody incubations were carried out in a humidity chamber for 1 h at 37°C. Nuclei were counterstained with Höechst 33342 (Molecular Probes, Cat. H-21492) dye or propidium iodide 0.2 µg/mL depending on the microscope used for visualizing, i.e., a Nikon optical microscope (Nikon eclipse TE2000-U, USA) or a Confocal Olympus FLuoview 1000 microscope (Olympus), respectively. Slides were mounted in Aqueous Mounting Medium with fluorescence tracers (Fluor Safe, Calbiochem, Cat. 345789).

### Western blotting

Whole protein extracts of JEG-3 and CTB cells were prepared in 5X Laemmli buffer containing 60 mM Tris-HCl pH 6.8, 10% glycerol, 2% sodium dodecyl sulphate, 1% 2-β-mercaptoethanol and 0.002% bromophenol blue. Total protein samples were separated on a 10% SDS-PAGE, and proteins were transferred to a nitrocellulose Hybond-ECL (Amersham Bioscience). The membranes were blocked in 5% non-fat milk in TBS (20 mM Tris-HCl, 150 mM NaCl pH 7.8), supplemented with 0.1% Tween-20 (TBS-T) 1 h at room temperature or overnight at 4°C according to the antibody used. Blots were incubated with primary antibodies diluted in 5% non-fat milk in TBS-T for 1 h at room temperature, except for the anti-Zf9 antibody which was incubated overnight at 4°C. The following antibodies were used: rabbit polyclonal anti-KLF6 (anti-Zf9, R-173, Santa Cruz Biotech.; 1∶750), rabbit polyclonal anti-PSG (anti β-1-glycoprotein, A0131, Dako;1∶1000), rabbit polyclonal anti-human chorionic gonadotropin (hCG; A0231, 1∶2000, Dako), rabbit polyclonal anti-GCM1 (AB2, Sigma-Aldrich; 1∶3000), mouse monoclonal anti-α-tubulin (Sigma–Aldrich; 1∶2000). After washing, the blots were incubated with horseradish peroxidase-conjugated donkey anti-rabbit or sheep anti-mouse IgG secondary antibodies (Amersham Bioscience; 1∶5000) in TBS-T, at room temperature for 1 h. Protein-antibody complexes were visualized using an enhanced chemiluminescence detection system (SuperSignalWest Pico; Pierce) and exposed to Kodak T-Mat G/RA film. Ponceau staining (0.2% Ponceau, 3% tricloroactic acid, 3% sulfosalicilic acid) was used to verify protein transference from gel to nitrocellulose membrane.

### Quantitative real time RT-PCR and semiquantitative RT-PCR

Total RNA was extracted from cultured cells at the indicated times using Trizol Reagent (Invitrogen). One microgram of total RNA was reverse-transcribed in a total volume of 20 µl using random primers (Invitrogen) and 50 U M-MLV reverse transcriptase (Promega Corp.).

Primers were manually designed with the assistance of the Netprimer software (PREMIER Biosoft International, Palo Alto, CA), UCSC In-Silico PCR (UCSC Genome Browser website) and Primer Express software (ABI, Applied Biosystems). Primer sequences were compared against the human genomic and transcript data base with the BLAST program [44] at the NCBI Web site. KLF6-specific primers (*KLF6-882 For* and *KLF6-960 Rev*) located in exons 2 and 3 of the human KLF6 gene [15] were designed in order to amplify only the wild type or full length KLF6 transcript. A different pair of primers (*KLF6-*F701 and *sv1FLF6-*R807) was designed to amplify the KLF6-SV1 splice variant through conventional RT-PCR.

Specific transcripts were quantified by real time qRT-PCR (ABI 7500 Sequence Detection System, Applied Biosystems) using the Sequence Detection Software v1.4. Experiments were performed using 1x SYBR Green PCR Master Mix (Applied Biosystems). Primer sequences and concentrations are indicated in Table 1. The cycling conditions included a hot start at 95°C for 10 min, followed by 40 cycles at 95°C for 15 s and 60°C for 1 min. Specificity was verified by melting curve analysis and agarose gel electrophoresis. Fold change in gene expression was calculated according to the 2^−ΔΔCt^ method [45]. Each sample was analyzed in triplicate. No amplification was observed in PCR reactions containing water or RNA samples incubated without reverse transcriptase during cDNA synthesis as template.

**Table 1:** Primer sequences and concentrations used in RT-PCR assays.

Gene/accession number	Primer name	Sequence (5′-3′)	nM
***KLF6***	*KLF6-882 For*	CAC CAA AAG CTC CCA CTT GAA	200
NM_001300	*KLF6-960 Rev*	CAC ACC CTT CCC ATG AGC AT	200
***KLF6sv1***	*KLF6-*F701	CCT CCA CGC CTC CAT CTT C	200
NR_027653	*sv1FLF6-*R807	ATC TGT AAG GCT TTT CTC CTT CC	200
***PSG*** * locus*	*PSGt For*	CCT CTC AGC CCC TCC CTG	100
*NC_000019.9*	*PSGt Rev*	GGC AAA TTG TGG ACA AGT AG AAG A	100
***GCM1***	*GCM1 For*	GAG GCA CGA CGG ACG CTT TAT ATT CAA	250
NM_003643	*GCM1 Rev*	TTG GAC GCC TTC CTG GAA A	250
***βhCG***	*βhCG-539 For*	GCT ACT GCC CCA CCA TGA CC	300
	*βhCG-632 Rev*	ATG GAC TCG AAG CGC ACA TC [47]	300
***PPIA***	*cyclo A For*	GTC AAC CCC ACC GTG TTC TT	100
NM_021130.3	*cyclo A Rev*	CTG CTG TCT TTG GGA CCT TGT	100

Semiquantitative RT-PCR of PSG and βhCG transcripts were performed in CTB cultures in order to confirm cell differentiation as described [7].

### Plasmids and DNA transfections

JEG-3 cells were transiently transfected with the pXJ-41-KLF6 or the ΔZF1-3 expression vectors containing the human wild type full-length KLF6 or the three zinc-finger carboxy terminus-deleted cDNAs, respectively [36]. The empty vector (pXJ-41) was used in control transfections. Luciferase reporter constructs containing the 5′ regulatory region of human *βhCG5* gene, −3700βhCG5-luc and −345βhCG5-luc were kindly provided by Dr M Knöfler. Constructions of PB3-luc, PB5-luc, UB5-luc and UB5mut-luc plasmids have been previously described [7], [46]. PB3-luc and PB5-luc contain the promoter and proximal regulatory region of *PSG3* and *PSG5* genes from positions −613/−49 and −605/−49, respectively. UB5-luc contains the promoter region of *PSG5* (−251/−49) and UB5mut-luc has the CPE-sequence mutated from 5′ CCC CAC CCA T 3′ to 5′ CCC Cga tat c 3′.

To overexpress KLF6, JEG-3 cells were seeded at a density of 5×10^5^ cells per 6-well culture plate. After 24 h, cells were transfected with 4 µl of Lipofectamine 2000 reagent and 1500 ng of the KLF6 expression vector or the control empty vector. Similarly, for transactivation experiments JEG-3 cells were seeded at a density of 1×10^5^ per well in 24-well plates, cultured for 24 h and afterwards transfected with 1 µl of Lipofectamine 2000 reagent, 500 ng of each *PSG* or *βhCG5* reporter construct, up to 300 ng of the expression or empty vector and 25 ng of the *Renilla* normalizing vector. Forty-eight hours post-transfection, cells were harvested and protein extracts were prepared in 100 µl of the passive lysis buffer. As controls, cells were transfected with the promoter-less luciferase vector (pGL3 basic). Luciferase and *Renilla* activities were measured in 10 or 20 µl of protein extracts on a GloMax-Multi Detection System (Promega Corp.) using the Dual-Luciferase Reporter Assay System (Promega Corp.), according to the manufacturer's instructions. For each cell extract, luciferase activity was normalized to *Renilla* activity and expressed as relative light units (RLUs). Relative promoter activity was calculated as the RLUs of each promoter construct in the presence, relative to the absence, of the expression vector.

### Statistical analysis

Results are presented as mean ±SEM or as median and range of three to eight independent experiments performed in duplicates or triplicates. A one-sample t-test was used to determine whether experimental values were significantly different from fixed control values set as 1. Nonparametric ANOVA was performed on quantitative mRNA and protein expression levels of KLF6 in CTBs during the differentiation time course using Kruskal-Wallis one-way Analysis of variance (ANOVA) with the Dunn's multiple comparisons post-hoc test. Statistical analyses were performed using the GraphPad Prism 5.0 software and p-values <0.05 were considered statistically significant.
